# Musculoskeletal ultrasound discriminating camptodactyly-arthropathy-coxa vara-pericarditis syndrome and juvenile idiopathic arthritis

**DOI:** 10.3389/fped.2026.1829170

**Published:** 2026-06-04

**Authors:** Hatice Adigüzel Dundar, Faekah Gohar, Daniel Windschall

**Affiliations:** 1 Clinic of Pediatric and Adolescent Rheumatology, St.-Josef-Stift Sendenhorst, Northwest Germany Center for Rheumatology, Sendenhorst, Germany; 2 Behcet Uz Pediatric Diseases and Surgery Training and Research Hospital, Clinic of Pediatric Rheumatology, University of Health Sciences, Izmir, Türkiye; 3 Medical Faculty, Martin Luther University Halle-Wittenberg, Halle, Germany

**Keywords:** camptodactyly-arthropathy-coxa vara-pericarditis syndrome, differential diagnosis, juvenile idiopathic arthritis, musculoskeletal ultrasound, power Doppler

## Abstract

**Objective:**

Camptodactyly-arthropathy-coxa vara-pericarditis syndrome (CACP) and juvenile idiopathic arthritis (JIA) both feature joint effusions. This study aimed to identify preliminary MSUS-based criteria that may help discriminate children with CACP from those with polyarticular JIA.

**Methods:**

A retrospective multicentre analysis of MSUS images from children with genetically confirmed CACP or a diagnosis of active polyarticular JIA was performed. Joint and tendon findings were evaluated using qualitative B-mode (BM) and Doppler-mode (DM) assessment and graded semi-quantitatively (0–3) according to OMERACT definitions.

**Results:**

All four children with CACP showed symmetrical joint involvement, with effusion present in 46/46 scanned joints and no intrasynovial hypervascularity (median DM = 0). In the JIA cohort (*n* = 6) 16/42 joints had asymmetric effusions, of which 56% showed intrasynovial hypervascularity (median DM = 1). All 16/16 scanned tendons in the CACP group showed no DM signal, whereas hypervascularity was observed in 7/8 affected tendons in the JIA cohort. Hyperechoic foci, suggestive of increased synovial fluid viscosity, were present in 18/46 (39%) affected joints in the CACP group compared to 1/16 (6%) in the JIA cohort.

**Conclusion:**

CACP is characterised by symmetrical large-joint effusions with hyperechoic foci and absence of intrasynovial DM signal, in contrast to inflammatory findings in JIA. These features may support early differentiation of CACP from JIA and therefore prompt earlier specific diagnostics, including genetic testing.

## Highlights

- Ultrasound features, including the Doppler assessment, may help differentiate CACP from JIA
- Multiple large, symmetrical hyperviscous joint and tendon sheath effusions were commonly observed in patients with CACP
- The absence of intrasynovial hypervascularity in DM may raise clinical suspicion for CACP compared to JIA joints and tendons

## Introduction

Camptodactyly-arthropathy-coxa vara-pericarditis syndrome (CACP) is an autosomal recessive genetic syndrome caused by mutation of the proteoglycan 4 (PRG4) gene which encodes the protein PRG4/lubricin. Lubricin is predominantly produced by synovial cells, but is also expressed by superficial zone chondrocytes, where it regulates synovial cell growth. It is additionally secreted by pericardial cells, where it exerts a lubricating effect. The absence of lubricin is therefore associated with hyperviscous joint effusions, synovial hyperplasia and articular surface damage. Within tendon sheaths, this absence also causes tissue damage with matrix remodelling and abnormal calcification. The most frequent characteristics of camptodactyly, progressive coxa vara and premature arthropathy without inflammation and non-inflammatory pericarditis have led to the naming of the syndrome (1, 2). CACP is therefore an important differential diagnosis of juvenile idiopathic arthritis (JIA) due to the presence of the peripheral joint arthropathy. Yilmaz et al. reported half of patients with CACP referred for genetic evaluation had received an initial diagnosis of JIA (3). Early differentiation of CACP from JIA is therefore vital, as the typical immune-modulating treatments used for JIA have side effects, are costly and ineffective in CACP (4, 5).

Radiographs, which are not routinely performed for JIA, might identify findings such as coxa-vara that could alert the clinician to a possible diagnosis of CACP. In JIA, musculoskeletal ultrasound (MSUS) is typically the imaging modality of choice for the identification of joint effusions and synovial hypertrophy (best seen in B-Mode, BM) and for intrasynovial hypervascularity (identified with power Doppler, PD), though MRI remains the gold standard (6, 7). There is a paucity of data describing musculoskeletal ultrasound findings in patients with CACP syndrome, particularly in comparison with inflammatory arthropathies such as JIA (3). Clinical series have consistently reported that CACP presents with symmetrical large-joint arthropathy, normal inflammatory markers, and frequent misdiagnosis as JIA (8). In contrast, the available literature on MSUS findings in CACP remains limited, consisting mainly of descriptive reports and small case series. This has been highlighted in recent literature, which emphasises key distinguishing features such as the absence of DM signal and non-inflammatory synovial proliferation (9). However, these observations have not been based on systematic multi-joint and tendon assessments or on direct comparative analyses with JIA. This study was therefore designed to systematically evaluate MSUS findings in children with CACP and compare these with those observed in children with active polyarticular JIA, with the aim of identifying preliminary imaging patterns to inform future multicentre studies.

## Materials and methods

This retrospective multicentre pilot study was conducted to evaluate MSUS findings in children with genetically confirmed CACP and to compare these with findings in children with active polyarticular JIA. The study was performed using routinely collected clinical and imaging data across participating paediatric rheumatology centres. For the JIA cohort, patients presenting in flare between 11 January 2024 and 1 February 2024 in one center were included. The study was designed as an exploratory, hypothesis-generating analysis, and findings should be interpreted within this context. Ethical considerations are described below.

### Literature review

A systematic review of the literature was performed using predefined search terms in the PubMed/MEDLINE and Embase databases, restricted to English-language publications prior to April 2026. The search terms included “Camptodactyly-arthropathy-coxa vara-pericarditis syndrome”, “Camptodactyly arthropathy coxa vara”, “Camptodactyly-arthropathy-coxa vara-pericarditis syndrome and JIA”, and “ultrasound features of Camptodactyly-arthropathy-coxa vara-pericarditis syndrome and JIA”. Identified articles were screened to confirm the inclusion of patients with genetically confirmed CACP syndrome. Eligible publications were subsequently reviewed to determine study type, patient population (CACP and/or JIA), and whether musculoskeletal ultrasound or other imaging findings were reported. Search terms were selected based on disease nomenclature, commonly used clinical descriptors and previously published terminology, and were adapted to each database using controlled vocabulary where applicable.

The literature search strategy was designed to capture all relevant publications reporting clinical or imaging findings in patients with genetically confirmed CACP syndrome. PubMed/MEDLINE and Embase were selected as they comprehensively index biomedical and imaging-focused journals, which are the primary sources for rare disease case reports and case series. Given the rarity of CACP syndrome, the majority of relevant publications are indexed within these databases. Eligible article types included case reports, case series and observational studies describing clinical, imaging or genetic findings in patients with CACP. Although additional databases such as Web of Science or Scopus could have been included, their omission was considered unlikely to significantly alter the results of the review.

### Patients

Children with CACP were identified by computer search from the paediatric rheumatology centres of the authors. Children with JIA, restricted to those with active seronegative polyarthritis were identified by computer search of recent admissions by the study secretary blinded to the study aims. Patients with polyarticular forms of JIA were selected, as the pattern of joint involvement in CACP is characterised by predominantly multiple joint involvement, more closely resembling polyarthritis than oligoarthritis. All patients fulfilling the inclusion diagnosis for JIA, without other musculoskeletal or inflammatory diagnosis who presented in acute flare between 11.01.2024–01.02.2024 were included. The JIA cohort was not intended to represent the full spectrum of JIA, but to provide a clinically relevant comparator group, with the chosen JIA subgroup diagnosed according to ILAR criteria reflecting the typical inflammatory ultrasound findings encountered and confused with CACP in routine practice (7, 10, 11). It was also not the purpose of this study to specifically power the examination findings for the analysis of MSUS features in children with JIA, as this has been widely published, but rather to use the findings from a consecutive sample of patients with polyarthritis as a comparison group for CACP.

A retrospective case analysis including a review of the history and clinical findings from the patient notes and clinic letters as well as MSUS-images was performed. As patients with JIA are typically in remission between flares, whereas CACP is characterised by chronic musculoskeletal involvement, the most recent clinical assessment was included for the CACP group and the most recent flare presentation for the JIA cohort. The demographic and clinical data evaluated included diagnosis, treatment exposure, and laboratory findings. Treatment status at the time of ultrasound assessment was recorded for all patients.

### Musculoskeletal ultrasound

Data analysis was performed by three paediatric rheumatologists, each with a minimum of five years’ experience in paediatric MSUS. The MSUS data included documentation of the joints and tendons examined and the presence of any pathological findings.

MSUS examinations were performed as part of routine clinical care across the participating centres. Inter-observer reliability was assessed using Fleiss’ kappa statistics, as described below.

B-mode (BM) images were evaluated for the presence of effusion, synovial thickening, or tenosynovitis. If effusion was present, it was further categorised according to echogenicity (anechoic to hyperechoic) and echostructure (homogeneous to inhomogeneous). Doppler-mode (DM) images were evaluated for the presence and grade of hypervascularity within the synovial tissue.

BM and DM views were evaluated using a semi-quantitative score based on grading from 0 to 3 according to the internationally agreed synovitis score from the Paediatric OMERACT group (12). The BM grades were scored as follows: grade 0 = no effusion, grade 1 = mild effusion and/or synovial hypertrophy, grade 2 = effusion and/or synovial hypertrophy leading to a convex shape of the recess, grade 3 = large effusion and/or synovial hypertrophy. DM images were each graded as follows: grade 0 = no intrasynovial DM signals, grade 1 = few dots of synovial DM signals, grade 2 = confluent DM signals in less than 30% of visible synovial tissue and grade 3 = confluent DM signals in more than 30% of visible synovial tissue. BM or DM positivity was defined as a score of ≥1.

To assess inter-observer reliability, a subset of MSUS images was independently evaluated by the three paediatric rheumatologists. Agreement was quantified using Fleiss’ kappa statistics for BM and DM grades, as well as for BM and DM positivity. Kappa values were interpreted according to standard benchmarks (13) (Table 3).

Descriptive statistical analysis was performed to summarise the clinical, laboratory, and sonographic data. Categorical variables were presented as frequencies and percentages, while continuous variables were reported as mean, median, standard deviation, minimum and maximum values.

Given the exploratory and hypothesis-generating design of this pilot study, the analysis was intentionally restricted to descriptive evaluation of MSUS findings. Formal statistical comparisons were not performed, as the study was not designed or powered to test predefined hypotheses, but rather to identify potential imaging patterns that may inform future larger-scale studies.

## Results

### Literature review

A systematic literature review identified 38 studies reporting patients with genetically confirmed CACP syndrome, comprising a total of 178 individuals. Most publications were case reports or small case series, and only two studies included a comparator group with JIA. A detailed summary of the included studies is provided in Supplementary Table S1.

Clinical series have consistently described CACP as a non-inflammatory arthropathy characterised by early-onset camptodactyly, symmetrical large-joint involvement, normal inflammatory markers, and frequent misdiagnosis as JIA. In a recent case series including 13 patients, large joint arthropathy affecting the wrists, ankles, elbows, and knees was observed in the majority of patients, with inflammatory markers remaining within normal ranges, further supporting the non-inflammatory nature of the disease (8).

Only a limited number of studies have reported MSUS findings in children with CACP. Furness et al. reported moderate effusion in the hip and knee joints in a child with CACP as assessed by MSUS (14). Similarly, Ciullini Mannurita et al. demonstrated the presence of joint effusion and synovial hyperplasia in large joints, although these findings were not derived from systematic ultrasound assessment (15). Albuhairan et al. described ultrasound examination with colour Doppler in a subset of their cohort, including interphalangeal (IP), metacarpophalangeal (MCP), and wrist joints, demonstrating synovial thickening without detailed assessment of the presence or extent of DM signal (16).

In a comparative study, Al-Mutairi et al. evaluated wrist and second and third MCP joints and reported synovial proliferation with normal DM signal in children with CACP, in contrast to increased hypervascularity observed in a subset of children with JIA (17). Earlier studies have similarly suggested synovial proliferation in the absence of increased vascularity as a distinguishing feature of CACP (18).

More recently, a narrative review has synthesised the available evidence and emphasised the role of MSUS in differentiating CACP from inflammatory arthritis, particularly highlighting the absence of DM signal, the presence of non-inflammatory synovial proliferation, and the description of hyperechoic intra-articular foci (9). However, despite these observations, the current literature remains limited by small sample sizes, lack of systematic multi-joint and tendon assessment, and minimal direct comparison with JIA cohort.

### Patients with CACP syndrome

Four children with CACP in two centres were identified. The symptoms started at a mean age of 7 ± 4 (SD) months (range 3-13) and a diagnosis of CACP was made at mean age 46 (16-108) months, with a mean delay in diagnosis of 39 (3-105) months. All children with CACP described joint and tendon swelling as their first symptoms. None had morning stiffness. The age at first diagnosis varied from 16 to 108 months. Two patients are siblings. The older sibling had previously been diagnosed with JIA with inadequate response to multiple therapies, including biologics, which raised suspicion for CACP when the younger sibling presented with a similar clinical history. All four patients developed camptodactyly, arthropathy and coxa vara and none had developed pericarditis to date. Pathogenic gene mutations confirming CACP were present in the four children with CACP, with compound heterozygous mutations in the two siblings and homozygous mutations in the other two children. The demographic and clinical characteristics of the CACP and JIA cohorts are summarised in Table 1.

**Table 1:** Demographic and clinical characteristics of children with CACP and JIA.

Characteristic	CACP, *n* = 4	JIA, *n* = 6
Age at study enrolment, years, mean ± SD (min-max)	7.3 ± 2.0 (4.5–9.0)	7.2 ± 4.3 (2.3–14.5)
- at first symptom (mth), mean ± SD (min-max)	7 ± 4 (3–13)	50 ± 23 (12–144)
- at first diagnosis (mth), mean ± SD (min-max)	46 ± 42 (16–108)	76 ± 59 (12–168)
- delay in diagnosis (mth), mean ± SD (min-max)	39 ± 45 (3–105)	26 ± 56 (0–144)
ANA positive, *n* (%)	1 (25%)	3 (50%)
RF positive, *n* (%)	0 (0)	0 (0)
Elevated CRP, *n* (%)	0 (0)	0 (0)
ESR (mm/h) mean ± SD (min-max)	12 ± 5 (5–16)	23 ± 12 (8–43)
Medication use, *n* (%) at any time		
Methotrexate use	1 (25)	6 (100)
Biologic therapy use	1 (25)	4 (67)
Imaging pathology at any time, patient *n* (%):		
Camptodactyly	4 (100%)	0 (0)
Coxa vara	4 (100%)	0 (0)
Pericarditis	0	0
Osteopenia in (x-Ray)	3 (75%)	0 (0)
Synovial hypertrophy (MRI)	3/3 (100%)	2/3 (66.7)
Bone erosions (MRI)	0 (0)	0 (0)

ANA, anti-nuclear antibody; CRP, C-reactive protein; ESR, erythrocyte sedimentation rate; MRI, magnetic resonance imaging; RF, rheumatoid factor; SD, standard deviation. Age is presented in years unless otherwise specified; early disease variables are reported in months.

### Patients with JIA

Children with JIA had a diagnosis of seronegative polyarthritis (*n* = 6) see Table 1. Five (83%) were female and three (50%) were ANA positive. Children presented with their first symptoms at a mean age of 4 years (range 1-12) and received their diagnosis at a mean age of 6 years (range 1-14). Five out of six had morning stiffness at onset. Three (50%) had a positive family history for an unspecified rheumatological disease, though not in first-degree relatives. All patients in the JIA cohort had previous or current methotrexate use while 4/6 were treated with biological therapies. At the time of ultrasound assessment, all patients in the JIA cohort were under treatment for JIA. In the CACP group, only one patient had received prior treatment, whereas three had not received any treatment prior to imaging.

### Musculoskeletal ultrasound features: CACP vs. JIA

The involvement of eight joint regions (shoulders, elbows, wrists, hips, knees, ankles, fingers and toes) and five tendon regions (shoulders, elbows, wrists, hands and feet) were analysed by retrospective review of the MSUS images. In total, 46 joints and 16 tendons for the CACP group and 42 joints and 20 tendons for the JIA cohort had been examined with MSUS (Table 2). All patients in both disease groups had joint involvement, with pathology detected in 46/46 scanned joints in the CACP group and 16/42 scanned joints in the JIA cohort. All 16/16 scanned tendons in the CACP group and 8/20 scanned tendons in the JIA cohort showed abnormalities, corresponding to involvement in all patients in the CACP group (4/4) and in 3/6 patients in the JIA cohort.

**Table 2:** Ultrasound features in CACP and JIA patients.

Anatomical region	Affected/Scanned	BM Grade (0–3) median (range)	DM Grade (0–3) median (range)	Effusion echogenicity			Effusion homogenous	Hyperechoic Foci present		Affected/Scanned, n	BM Grade (0–3) median (range)	DM Grade (0–3) median (range)
				Anechoic	Hypoechoic	Hyperechoic						
Joint n									Tendon n			
Scanned joints and tendons, CACP (*n* = 4 patients)												
Shoulder	6/6	3 (3–3)	0	0	4	2	2/6	6/6	Shoulder (Biceps)	6/6	3 (2–3)	0
Elbow	8/8	3 (3–3)	0	6	0	2	4/8	6/8	Elbow (extensors)	2/2	2 (2–2)	0
Wrist	8/8	2 (1–3)	0	8	0	0	8/8	8/8	Wrist (extensors)	4/4	2 (1–2)	0
Hip	8/8	3 (2–3)	0	2	6	0	4/8	4/8	Posterior Tibialis	2/2	2 (2–2)	0
Knee	8/8	3 (1–3)	0	4	4	0	4/8	2/8	Flexor digitorum	2/2	1 (n/a)	0
Ankle (Upper)	8/8	3 (2–3)	0	4	4	0	6/8	0/8				
Total affected, *n* (%)	46/46 (100)	n/a	n/a	24/46	18/46	4/46	28/46 (61)	18/46 (39)		16/16 (100)	n/a	n/a
Median	n/a	3	0						n/a		2	0
Scanned joints and tendons, JIA (*n* = 6 patients)												
Shoulder	0/2	n/a*	n/a*				n/a*	n/a*	Shoulder (Biceps)	0/2	n/a*	n/a*
Elbow	2/2	3 (2–3)	1 (0–1)	0	2	0	0/2	0/2	Elbow (extensors)	0/0	n/a*	n/a*
Wrist	2/4	2 (2–2)	3 (3–3)	0	2	0	2/2	0/2	Wrist (extensors)	2/4	2 (2–2)	2 (2–2)
Hip	3/10	1 (1–2)	0	0	2	1	0/3	0/3	Posterior Tibialis	5/8	2 (2–2)	2 (0–3)
Knee	5/12	2 (2–3)	2 (2–3)	0	5	0	2/5	1/5	Flexor digitorum	1/6	2 (n/a)	3 (n/a)
Ankle (Upper)	4/12	3 (1–3)	0 (0–1)	0	4	0	1/4	0/4				
Total affected, *n* (%)	16/42 (38)	n/a	n/a				5/16 (31)	1/16 (6)		8/20 (40)	n/a	n/a
Median	n/a	2 (2)	1							n/a	2	2

BM, B-mode; DM, Doppler mode; CACP, Camptodactyly-arthropathy-coxa vara-pericarditis syndrome; JIA, Juvenile idiopathic arthritis; n/a, not applicable, *areas were not routinely scanned if no pain, swelling or limitation of range of motion was present. .

All patients in the CACP group had simultaneous bilateral involvement of the shoulder and elbows as well as the large joints of the lower extremities, which was a combination not seen in the JIA cohort. Biceps tendinitis was present in 6/6 tendons tested in the CACP group, and in none of the evaluated tendons in the JIA cohort (Table 2).

**Figure 1:**
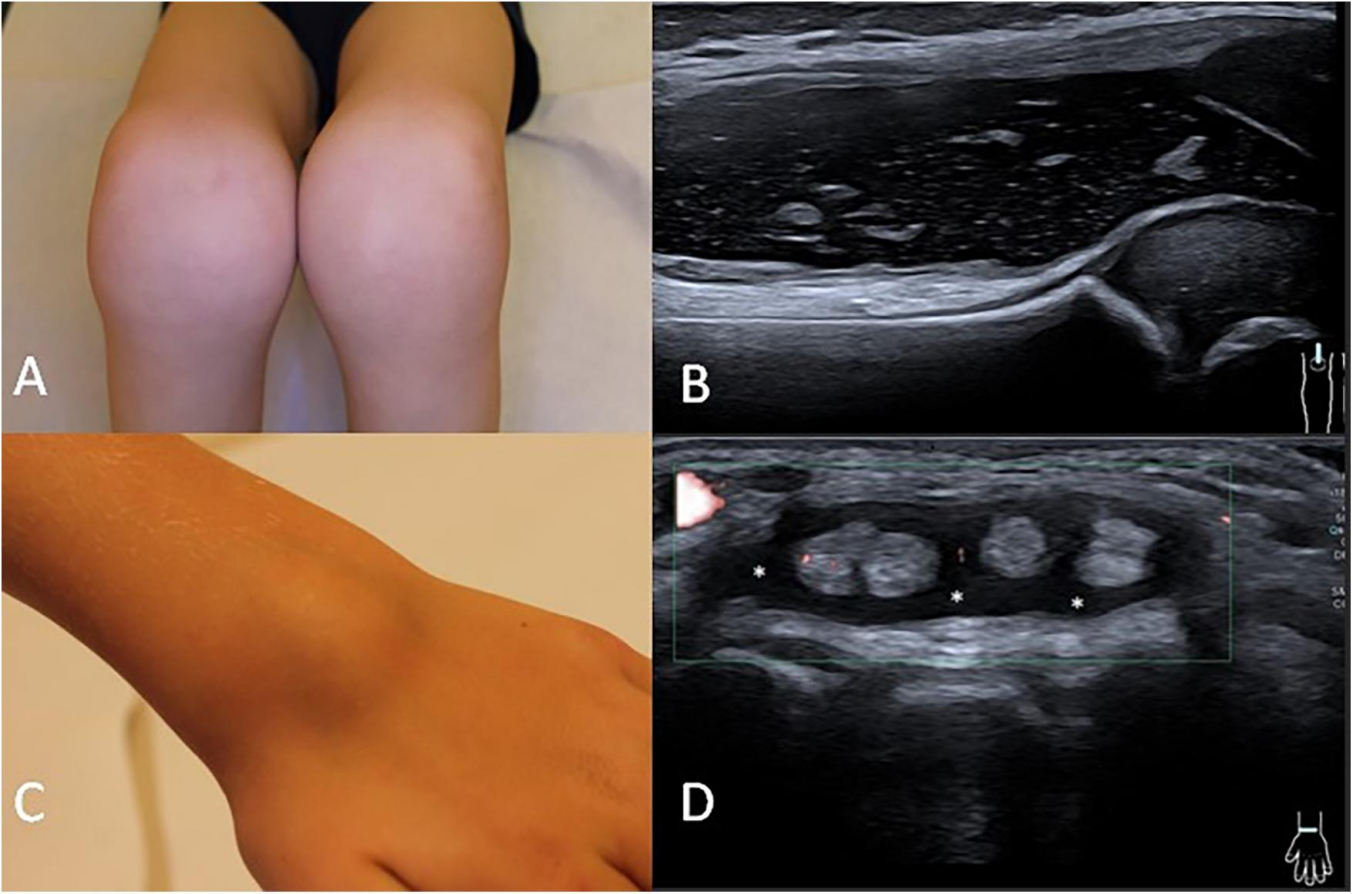
The panel shows clinical and ultrasound images from two patients with CACP. Panels A and C demostrate clinical features of symmetrical knee joint swelling and swelling of the wrist extensor tendon region, respectively. Panels B and D show the corresponding ultrasound images. Panel B demonstrates a large effusion (BM grade 3) with hyperechoic foci within an otherwise anechoic effusion and absence of synovial hypertrophy in the suprapatellar longitudinal view. Panel D shows an anechoic effusion of the extensor tendon sheath in the transverse view. BM, B-mode; CACP, Camptodactyly-arthropathy-coxa vara-pericarditis syndrome; CI, confidence interval; JIA, juvenile idiopathic arthritis.

Effusions were present in at least one joint in all patients with CACP and JIA. BM grading of MSUS findings in the affected joints was a median grade 3 in the CACP group and a median 1 in the JIA cohort. In tendons, BM grading was a median grade 2 for all affected tendons in both the CACP and the JIA group. DM positivity (DM grading≥1)/hypervascularity was present in none of the 46 affected joints or 16 affected tendons scanned in the CACP group. In the JIA cohort, DM positivity/hypervascularity was present in 9/16 affected joints (56%) and 7/8 affected tendons (88%). DM grading was a median 0 in affected joints and tendons of children with CACP. In the JIA cohort DM median was 1 in affected joints and 2 in affected tendons. Figure 1 shows a clinical and corresponding ultrasound example of knee and wrist extensor tendon involvement in two patients with CACP.

**Figure 2:**
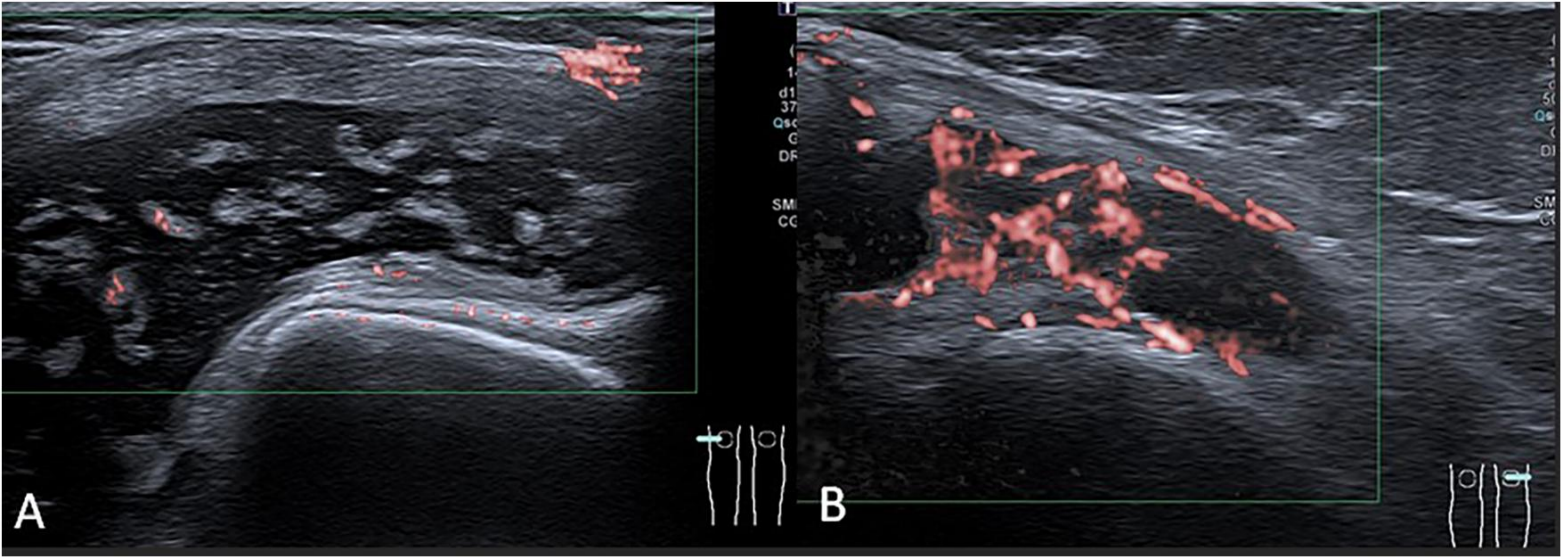
Ultrasound images of the lateral parapatellar recess of the knee in a patient with CACP **(A)** and a patient with JIA **(B)** are shown. Both images demonstrate large effusions (OMERACT BM grade 3). Panel A shows synovial hypertrophy and multiple hyperechoic foci suggestive of increased viscosity, whereas Panel B shows synovial hypertrophy without the hyperechoic foci. In addition, marked intrasynovial hypervascularity (DM grade 3) is present in Panel B but is absent in Panel A. BM, B-mode; CACP, Camptodactyly-arthropathy-coxa vara-pericarditis syndrome; CI, confidence interval; DM, Doppler mode; JIA, juvenile idiopathic arthritis.

Inhomogeneous effusions with hyperechoic foci were found in 18/46 (39%) of affected CACP joints and 1/16 (6%) of affected JIA joints. The CACP group was more likely to have anechoic or homogeneous effusions compared to the JIA cohort. Figure 2 shows ultrasound images of the lateral parapatellar recess of a knee joint from an included CACP and JIA patient where large effusions with BM grade 3 can be seen with multiple hyperechoic foci in the joint of the CACP patient. Additionally, a DM grade of 3 is evident in the knee joint of the child with JIA, whilst no DM signals could be detected in the patient with CACP. No patients in either group had visible bone erosions or cartilage damage as evaluated by MSUS, MRI or plain radiographs.

Inter-observer reliability analysis demonstrated substantial to almost perfect agreement for BM and DM grades and positivity, with Fleiss’ kappa values ranging from 0.673 to 1.0 (Table 3).

**Table 3:** Inter-observer reliability assessed by fleiss’ kappa.

Parameter	Kappa	95% CI	p
BM grades	0.747	0.544–0.951	<0.001
BM positivity	0.819	0.477–1.160	<0.001
DM grades	0.673	0.435–0.912	<0.001
DM positivity	1.0	0.623–1.370	<0.001

BM and DM grades were assessed using a semi-quantitative scoring system (grade 0–3) based on paediatric OMERACT synovitis definitions. Positivity was defined as a score ≥1, with grade 0 considered negative.

BM, B-mode; DM, Doppler mode; CI, confidence interval.

## Discussion

This study describes a distinct MSUS pattern in children with CACP syndrome, characterised by symmetrical large-joint effusions with hyperechoic foci and absence of intrasynovial DM hypervascularity, in contrast to the findings observed in children with active polyarticular juvenile idiopathic arthritis. In addition, a review of the existing literature on ultrasound findings in CACP was performed to contextualise the findings of this preliminary investigation, designed to inform a planned multicentre study within the Imaging Working Party of the Paediatric Rheumatology European Society (PReS).

Whilst the included patient sample is small, it reflects the rarity of the CACP diagnosis. The MSUS findings of the JIA cohort are well known and already published (6, 7, 12). However, six children with active polyarticular JIA were included to allow comparison under the same MSUS setting and equipment as used for three of the four CACP patients, as one patient was assessed at a different centre. As the existing literature on musculoskeletal ultrasound findings in CACP syndrome is sparse, this dataset is informative, given the number of joints and tendons analysed. The literature review revealed few studies on CACP syndrome included MSUS data (5/35 articles) and of these studies, one was a review article which summarised the presence of synovial proliferation in the absence of increased vascularity (19). Of the remaining four clinical studies, only one study included a comparison of findings in CACP and JIA patients. The comparative study by Al-Mutairi et al. similarly reported differences in Doppler findings between CACP and JIA (17). In that study, increased vascularity was observed in a subset of children with JIA. In contrast, hypervascularity was detected in 56% of affected joints in the JIA cohort in the present study, reinforcing the discriminatory value of Doppler findings. Albuhairan et al. also reported limited and non-systematic ultrasound assessment, without detailed evaluation of effusion characteristics or Doppler-defined hypervascularity (16).

No MSUS findings in tendons were described in the identified studies. Furness et al. described the MSUS findings of effusion in hip and knee joints in their single CACP patient (14). Ciullini Mannurita et al. also included only large joints in their study, and described findings of joint effusion with synovial hyperplasia in the majority of patients although the focus of the paper was genetic analysis (15). Therefore, this study expands on the existing literature by providing a systematic multi-joint and tendon assessment across multiple anatomical regions, which has not been previously reported.

Articular manifestations are predominant in children with CACP syndrome, leading to frequent misdiagnosis as juvenile arthritis. Large joints are most commonly affected, e.g., the wrists, knees, elbows, shoulders, hips and ankles with swelling, limited motion, and flexion contractures, representing non-inflammatory arthritis and therefore morning stiffness is usually absent. The CACP group included here had these characteristics, as well as a typical absence of morning stiffness, camptodactyly and coxa vara. However, none had yet developed pericarditis, which is a feature reported in 10%-22% of patients in the literature (20–22).

MSUS is the most frequently used diagnostic method in paediatric rheumatology (7). It has the advantage of being widely available, easily accessible, non-invasive, fast and allows multiple joints to be examined without radiation exposure or sedation. In this cohort, BM positivity of shoulders, elbows and the lower large joints (hips, knees and ankles) appeared symmetrically in the CACP group, whereas this pattern was not seen in the examined JIA cohort. This could also be due to the JIA sample which did not include any patients with rheumatoid factor positive arthritis, which typically presents with symmetrical involvement of both large and small joints. However, the BM grading was higher in CACP vs. JIA, indicating significant synovial thickening/proliferation with effusion. Synovial tissue in the CACP group also had an absence of hypervascularity as indicated by an absence of DM signal, which was also in stark contrast to the DM findings in joints of the JIA cohort. Whilst effusions in the CACP group tended to be either hypoechoic or anechoic, effusions in the JIA cohort were almost exclusively hypoechoic. An additional finding in the CACP group was the presence of hyperechoic foci in effusions in 18/46 of the affected scanned joints in CACP but only in 1/16 JIA joints. In CACP a deficiency of lubricin results in pathological changes within the joint, including abnormal protein deposition and calcification and may also lead to increased synovial fluid viscosity. These changes may contribute to the formation of dense intra-articular structures, observed as hyperechoic foci in the CACP group. These findings may reflect the presence of dense intra-articular material, such as proteinaceous content or other highly reflective structures. Such features are more likely to be recognised with use of the newer and most sensitive ultrasonography machines (2, 23).

Marked symmetrical tenosynovitis involving multiple tendon regions, including shoulder (biceps), elbow extensors, wrist extensors, posterior tibialis, and flexor digitorum tendons, was observed in the CACP group, with numerically higher median BM values compared to the JIA cohort. In contrast, as with the joint findings, DM positivity did not occur in CACP tendons but was present in affected tendons in the JIA group. In this study, patients in the JIA cohort had already received treatment at the time of inclusion. Newly diagnosed patients with JIA were not used as the comparison group, as the CACP group was also not newly diagnosed. In addition, the subtype of JIA was pre-selected to most closely match the pattern of joint involvement in CACP. Patients in the JIA cohort presenting with flare within the same month were included to limit potential selection bias.

The high inter-observer agreement further supports the reliability and reproducibility of the ultrasound assessments.

This study has several important limitations that should be considered when interpreting the findings. Firstly, patients were not assessed at initial disease presentation, which may limit interpretation of early imaging features. Secondly, patients in the JIA cohort had received prior treatment at the time of ultrasound assessment, whereas only one patient in the CACP group had received prior treatment. Prior exposure to corticosteroids, DMARDs or biologic therapies may therefore have influenced the observed findings, particularly DM signal. Thirdly, imaging acquisition and Doppler settings were not fully standardised across centres, which may have affected the sensitivity of detecting hypervascularity. Finally, the small sample size reflects the rarity of CACP and limits generalisability; however, the systematic multi-joint and tendon assessment provides a detailed exploratory dataset that offers clinically relevant insights and supports the rationale for future multicentre studies.

In summary, the use of BM and DM in MSUS examination in the CACP group was associated with symmetrical anechoic/hypoechoic effusions with synovial thickening in the large joints, in the absence of intrasynovial hypervascularity. Hyperechoic foci within joint effusions observed on BM were predominantly seen in CACP-affected joints and only rarely observed in the JIA cohort, suggesting a possible association with increased synovial fluid viscosity. Tendon involvement was also present in a symmetrical manner in the CACP group, without associated intrasynovial hypervascularity on DM.

The findings of the present study build upon recent literature suggesting that MSUS can assist in differentiating CACP from inflammatory arthritis. Previous reports have highlighted key features such as the absence of DM signal, non-inflammatory synovial proliferation, and the presence of hyperechoic intra-articular foci (9). However, the existing literature is largely based on descriptive reports and lacks systematic imaging assessment across multiple joints and tendon regions. In contrast, the present study provides a structured evaluation of both joint and tendon involvement, with direct comparison to a clinically relevant JIA cohort, thereby extending and strengthening these observations and offering a more comprehensive characterisation of the MSUS pattern in CACP.

Taken together, these findings define a clinically recognisable MSUS pattern that may facilitate earlier differentiation of CACP from inflammatory arthritis in clinical practice. In particular, the combination of symmetrical large-joint effusions, absence of DM signal, and the presence of hyperechoic intra-articular foci may serve as key imaging features raising suspicion for CACP. This is especially relevant in children initially diagnosed with JIA who show an atypical clinical course or suboptimal response to immunomodulatory therapy. Recognition of this pattern may prompt timely reconsideration of the diagnosis and support further evaluation, including genetic testing. These results provide a clinically applicable framework while also highlighting the need for validation in larger, prospective multicentre studies.

## Data Availability

The original contributions presented in the study are included in the article/Supplementary Material, further inquiries can be directed to the corresponding author.
