# Supplementary material for: Musculoskeletal ultrasound discriminating camptodactyly-arthropathy-coxa vara-pericarditis syndrome and juvenile idiopathic arthritis
Source: Front Pediatr. 2026 Jun 4;14:1829170. doi: 10.3389/fped.2026.1829170 (PMC13275454; doi:10.3389/fped.2026.1829170)
Supplement: Supplementary file 1 [file Datasheet1.pdf]

Supplement Table 1: Systematic literature review summary

Reference/Citation	Study type	Ultrasound performed? If yes, summary of results	Patients included with CACP	Patients included with JIA	Focus of the paper	Other Imaging described?
Alazami AM et al. Hum Mutat. (2006)[1]	Case Series	No	7 patients	0	Focus on genetics	n/a
Albuhairan et al. Semin Arthritis Rheum. (2013)[2]	Case series	MSUS of IPs, MCPs and wrists with color Doppler showed synovial thickening without synovitis.	22 patients	0	Clinical, laboratory, radiological and genetic findings	Bilateral hand MRI of one patient showed low intensity at T1 and high intensity on STIR and T2 indicating increased fluid around the extensor tendons of the 2–5 metacarpals and extended to the level of the wrist joint. There was no bony involvement.
Albtoush OM et al. Rofo. (2018)[3]	Case Report	Unknown, full text not available	1 patient	0	Shoulder joint involvement	
Al-Mayouf SM et al. Mol Imaging Radionucl Ther. (2017)[4]	Prospe ctive clinical trial	No	6 patients	0	Therapathic evaluation (Efficacy of Yttrium-90 radiosynovect omy)	MRI; moderate to severe knee joint effusion with thickened synovium
Al-Mayouf SM. Int J Pediatr Adolesc Med. (2018)[5]	Review	What distinguishes CACP syndrome from inflammatory arthritis is the presence of synovial proliferation with normal synovial vascularity on ultrasonography.	n/a	n/a (findings in JIA are discussed)	Features of non-inflammatory diseases that mimic JIA	XR; coxa vara, acetabular cysts
Al-Mutairi et al. Ann Paediatr Rheumatol. (2013)[6]	Resear ch article	US examination; 6 joints (wrist, 2nd and 3rd MCP joints bilaterally). In CACP patients synovial proliferation was reported to be more prevalent	5 patients	19 patients	Utility of ultrasonograp hy in CACP patients	

		(significant P-value) with a normal synovial vascularity on colour Doppler assessment versus the JIA patients. JIA patients were more likely to have joint effusion and bone erosions (not statistically different) .<br><br>*Full text not available				
Akawi NA et al. Birth Defects Res A Clin Mol Teratol. (2012)[7]	Case report	No	4 patients	0	One of them had congenital cataract<br><br>Genetics	n/a
Bahabri SA et al. Arthritis Rheum. (1998)[8]	Case Series	Unknown, full text not available	8 patients	0	Genetics	
Basit S et al. Arch Med Res. (2011)[9]	Case report	No	6 patients	0	Genetics	XR; flexion deformity of fingers and toes, coxa-vara, enlarged femoral head, enlargement of joint spaces, epiphyseal deformation, enlarged femoral head with short femoral neck and osteoporosis.
Bulutlar G et al. Arthritis Rheum. (1986)[10]	Brief report	No	4 patients	0	Clinical and laboratory findings	XR; coxa vara
Bağrul et al. Pediatr Rheumatol Online J. (2023)[11]	Case Series	No	2 patients	0	Genetics	XR; narrowed bilateral hip,knee,wrist,elbow joint space. Coxa vara and subcondral cysts.<br><br>MRI; chronic degenerative changes, coxa vara deformity, subchondral cysts, and synovitis in hip joints. Subchondral cysts on the humeral head and synovitis in the joint

						space and subscapular bursa in shoulder
Choi BR et al. J Korean Med Sci. (2004)[12]	Case Report	No	1 patient	0	Clinical, radiological, and pathological findings	XR; coxa vara in hip, flexion deformity in hands and feets<br><br>MRI; increased joint fluid with a thin rim-like enhancement surrounding the fluid-filled bursae in knees
Ciullini Mannurita S et al. Eur J Hum Genet. (2014)[13]	Research article	Arthropathy in large joints. Joint effusion with synovial hyperplasia (11/13 effusion, 12/13 synovial hyperplasia)	13 patients	0	Genetics	XR; joint erosions (6/13), coxa vara (11/13)
El-Garf A et al. J Rheumatol. (2003)[14]	Research article	No	10 patients	0	Clinical, radiological, and pathological findings	XR; camptodactyly and widening of hip joint space in all cases/ widening of knee joint space (80%).<br><br>MRI; various grades of joint effusion in all cases. The enhancement appeared thin, uniform, and rim-like in 7 cases/ homogenous in 2 cases
Emad, Y. et al. Joint bone spine. (2013)[15]	Case report	no	1 patient	0	Axial involvement of CACP	XR- fusiform swelling of the PIP, squaring of MCP, absence of erosive changes. coxa vara, flattening of femoral heads.<br><br>MRI; bilateral hypertrophic facet joint arthropathy at the level of L5/S1.<br><br>CT; bilateral L5/S1 facet joints ankylosis
Faivre L et al. Am J Med Genet. (2000)[16]	Case report and literature review	No	12 patients	0	Clinical variability and genetic homogeneity<br><br>Hip and spine involvement	XR; enlargement of the femoral head, short femoral neck, hypoplasia of iliac bones and osteoporosis. Coxa vara

						Spine abnormalities (cyphosis, lordosis, or scoliosis)
Furness, L et al. Pediatric rheumatology online journal (2022)[17]	Case Series	MSUS was performed in 1 patient. Single images, without Doppler assessment, of one knee and one hip joint indicate moderate effusion (BM 2-3) with minimal synovitis.	2 patients with CACP out of 4 with an initial diagnosis of JIA	0	Clinical features of a few diseases mimicking JIA	XR; coxa vara shallow acetabular, hands- periarticular osteopenia<br>MRI; bilateral coxa-vara, with short and broad femoral necks. Effusion and synovial thickening of bilateral knee and hip.
Hugosson C et al. Pediatr Radiol. (1994)[18]	Research article	No	5 patients	0	Radiological features in CACP	XR; Coxa vara, short broad femoral neck, acetebular cysts. Increased joint space, effusion, small iliac wings, flat and irregular femoral head. Flattened olecranon fossa and prominent radial head in elbow.<br>MRI; Fluid in joints penetrating into acetabular bone.
Johnson N et al. Rheumatol Int. (2021)[19]	Review	No	2 patients	0	Association of MVP and regurgitation, constructive pericarditis	XR; Bilateral coxa vara, short femoral neck, no erosions. Widening of the joint space in knees. Periarticular osteopenia in knees
Kakkar RM et al. J Clin Imaging Sci. (2013)[20]	Case report	no	1 patient	0	Synovial pathology- giant cell infiltration, with positive expression of CD68.	XR; smooth flattening of femoral heads with irregular acetabulae, coxa vara. Periarticular osteopenia, enlargement of the epiphyses, absence of joint erosions in knees.<br>MRI; bilateral hip joint effusions and intra-osseous acetabular herniations. rim enhancement of bilateral hip joint capsule (T1WI).

Kisla Ekinci RM et al. Mol Syndromol. (2021)[21]	Case Report	No	3 patients	0	Serum lubricin levels	XR; coxa vara and bilateral subchondral sclerosis and irregularity of acetabular. Loss in joint spaces of PIP<br><br>MRI; synovial thickening and effusion of bilateral large joints and acetebular cycts.
Laxer RM et al. Arthritis Rheum. (1986)[22]	Brief report	No	1 patient	0	Pathology of synovium and pericardium	XR; osteoporosis and soft tissue swelling in large joints, no periostitis, joint space narrowing, erosions
Madhusudan S et al. Scand J Rheumatol. (2016)[23]	Case report	no	1 patient	0	Clinical and radiographic findings	XR; bilateral coxa vara, smooth flattening of the femoral heads, irregular acetabular surface, and prominent radiolucent lesions within both acetabula.<br><br>MRI; bilateral coxa vara and bilateral hip joint effusion, with herniation of the joint fluid into the acetabulum, forming cavities.
Maniscalco V et al. Front Pediatr. (2022)[24]	Case Report and literature review	No	1 patient		Temporamandibular involvement, Therapathic evaluation	MRI; (hip,knee and ankle)-sinovial hyperplasia, joint effusion and bone erosions in both the coxofemoral joints and knees. (left TMJ)- intra-articular effusion and irregular surface on the condyle.
Marcelino J et al. Nat Genet. (1999)[25]	Literature Review	No	8 patients		To identify biological pathways of CACP	XR; . Subchondral bone cysts in the femoral head and acetabulum. Periarticular osteoporosis at the affected joins.
Murphy JM et al. J Arthroplast. (2012)[26]	Case report	No	2 patients		Total hip arthroplasty	XR; coxa vara in the bilateral hips with flattened femoral heads, joint-space narrowing, and subchondral sclerosis

Nandagopalan RS et al Indian J Med Res. (2014) [27]	Case report	No	3 patients		Genetics	XR- increased joint spaces in the large joints with periarticular osteopenia.
Offiah AC et al. AJR Am J Roentgenol. (2005) [28]	Research article	No	1 patient	Unclear in the methods, images from 5 patients shown	differentiation of CACP syndrome from juvenile idiopathic arthropathy.	XR; flattening of femoral head, presence of large acetabular cysts (benign radiolucent acetabular lesions), bilateral coxa vara, periarticular osteopenia, squaring of metacarpals and phalanges, absence of erosive change, loss of joint spaces of carpal and interphalangeal joints and camptodactyly<br><br>MRI with gadolinium; large bilateral joint effusions in large joints. Rim enhancement of joint capsule and of walls of interosseous herniations (T1WI of hips).
Patil DV et al. Indian Heart J. (2016)[29]	Case Report	No	2 patients		Constructive pericarditis	XR; short femur neck, flattened acetabulae, and nonerosive arthropathy without periarticular osteopenia
Peters B et al. Pediatr Rheumatol Online J. (2016)[30]	Case Report	Yes, (no further details provided)	1 patient		PLE caused by constrictive pericarditis	XR; coxa vara, peri-articular osteopenia and flattened metacarpal and phalangeal joints
Shashaani et al. Musculoskelet Disord. (2025) [31]	Case report	No	1 patient	0	Clinical presentation and genetic diagnosis	MRI; joint effusion and synovial thickening
Shayan, K et al. Pediatr Dev Pathol. (2005)[32]	Case report	no	3 patients	0	Synovial pathology	No
Singh et al. Clin Dysmorphol. (2024) [33]	Case series	No	13 patients	0	Clinical and genetic characteristics of CACP	XR; coxa vara, large joint involvement
Sparchez M. et al. Med Ultrason (2025) [34]	Review	MSUS; synovial proliferation with absence of Doppler signal;	n/a	n/a	Ultrasound differentiation of CACP and	MRI; supportive findings in selected cases

		non-inflammatory joint effusion; hyperechoic intra-articular foci described			inflammatory arthritis	
Taşar M et al. Turk J Pediatr. (2014)[35]	Case Report	No	1 patient	0	Pericardial involvement	Echocardiography- cardiac tamponade signs with pericardial effusion.
Vutukuru R et al. Indian J Med Res. (2016)[36]	Case report	No	1 patient	0	Clinical and radiographic findings	XR; acetabular cysts, increased joint space, flattened femoral heads, broadening of the femoral neck, and coxa vara.<br>MRI; multiple acetabular cysts.
Yilmaz S et al. Mol Genet Genomic Med. (2018)[37]	35 cases	No	35 patients	0	Genetics	XR; (pelvis)- narrowing acetabular space, irregularity of femoral capitis with aging, osteoporosis, short femoral neck, and coxa vara. (Wrist)- cystic radiolucent lesion
Zhang, J. M. et al. The Journal of rheumatology (2022)[38]	Case Series	No	4 patients	0	Clinical, laboratory, radiological and genetic findings	MRI; (right wrist)- multiple cystic and nodular areas with long T2 signals, indicating the presence of irregular effusion. (bilateral hip)- joint effusion and cyst formation.
Abbreviations: CACP; Camptodactyly-Arthropathy-Coxa Vara-Pericarditis, CT; computed tomography, IP; proximal interphalangeal, IP; interphalangeal, MCP; metacarpophalangeal, MRI; magnetic resonance image, MSUS; musculoskeletal ultrasound, MVP; mitral valve prolapse, n/a; not applicable, TMJ; temporomandibular joint, US; ultrasound, PLE; protein-losing enteropathy, STIR; short tau inversion recovery, XR; X-Ray (radyography), WI; weighted image						

Full reference list

1. Alazami AM, Al-Mayouf SM, Wyngaard CA, Meyer B. Novel PRG4 mutations underlie CACP in Saudi families. Hum Mutat. (2006) 27:213. doi: 10.1002/humu.9399
2. Albuhairan I, Al-Mayouf SM. Camptodactyly-arthropathy-coxa vara-pericarditis syndrome in Saudi Arabia: clinical and molecular genetic findings in 22 patients. Semin Arthritis Rheum. (2013) 43:292–6. doi: 10.1016/j.semarthrit.2012.11.004
3. Albtoush OM, Taib AA, Manzalawi KA, Mahafza WS. Camptodactyly-arthropathy-coxa vara-pericarditis syndrome with shoulder joint involvement: a case report with literature review. Rofo. (2018) 190:856–8. doi: 10.1055/s-0043-120765
4. Al-Mayouf SM, Almutairi N, Alismail K. Yttrium-90 radiosynovectomy in patients with

CACP syndrome. Mol Imaging Radionucl Ther. (2017) 26:33–7. doi: 10.4274/mirt.29484

5. Al-Mayouf SM. Noninflammatory disorders mimic juvenile idiopathic arthritis. Int J Pediatr Adolesc Med. (2018) 5:1–4. doi: 10.1016/j.ijpam.2018.01.004
6. Mutairi M, AlIsmeal K, Almulhem A, Al-Mayouf SM, Al-Mutairi M, Al-Suwaid A, et al. Utility of ultrasonography in children with camptodactyly-arthropathy-coxa vara-pericarditis syndrome. Ann Paediatr Rheumatol. (2013) 2:107–11. doi: 10.5455/apr.061820130509
7. Akawi NA, Ali BR, Al-Gazali L. A novel mutation in PRG4 gene underlying camptodactyly-arthropathy-coxa vara-pericarditis syndrome with possible expansion of the phenotype to include congenital cataract. Birth Defects Res A Clin Mol Teratol. (2012) 94:553–6. doi: 10.1002/bdra.23031
8. Bahabri SA, Suwairi WM, Laxer RM, Polinkovsky A, Dalaan AA, Warman ML, et al. The camptodactyly-arthropathy-coxa vara-pericarditis syndrome: clinical features and genetic mapping to human chromosome 1. Arthritis Rheum. (1998) 41:730–5. doi: 10.1002/1529-0131(199804)41:4
9. Basit S, Iqbal Z, Umicevic-Mirkov M, Ali N, Ahmad W, Ansar M, et al. A novel deletion mutation in proteoglycan-4 underlies camptodactyly-arthropathy-coxa-vara-pericarditis syndrome in a consanguineous Pakistani family. Arch Med Res. (2011) 42:110–4. doi: 10.1016/j.arcmed.2011.04.006
10. Bulutlar G, Yazici H, Ozdogan H, Schreuder I. A familial syndrome of pericarditis, arthritis, camptodactyly, and coxa vara. Arthritis Rheum. (1986) 29:436–8. doi: 10.1002/art.1780290321
11. Bağrul I, Ceylaner S, Yildiz YT, Kılıç SS, Çakan M, Kasapçopur Ö, et al. A novel mutation in the proteoglycan 4 gene causing CACP syndrome: two sisters report. Pediatr Rheumatol Online J. (2023) 21:8. doi: 10.1186/s12969-023-00793-z
12. Choi BR, Lim YH, Joo KB, Kim JH, Park HJ, Lee SJ, et al. Camptodactyly, arthropathy, coxa vara, pericarditis syndrome: a case report. J Korean Med Sci. (2004) 19:907–10. doi: 10.3346/jkms.2004.19.6.907
13. Mannurita SC, Vignoli M, Bianchi L, Cantarini L, Tani C, Frediani B, et al. CACP syndrome: identification of five novel mutations and of the first case of UPD in the largest European cohort. Eur J Hum Genet. (2014) 22:197–201. doi: 10.1038/ejhg.2013.123
14. El-Garf A, Mahmoud G, Gheith R, Abd El-Aaty G, Abd El-Aaty H. Camptodactyly, arthropathy, coxa vara, and pericarditis syndrome among Egyptians. J Rheumatol. (2003) 30:1081–6
15. Emad Y, Ragab Y, Khalifa M, Rasker JJ, El-Shaarawy N, Abou-Zeid A, et al. Axial involvement with facet joint arthropathy and bony ankylosis in camptodactyly-arthropathy-coxa vara-pericarditis syndrome. Joint Bone Spine. (2013) 80:520–2. doi: 10.1016/j.jbspin.2013.01.010
16. Faivre L, Prieur AM, Le Merrer M, Hayem F, Maroteaux P, Munnich A, et al. Clinical variability and genetic homogeneity of the camptodactyly-arthropathy-coxa vara-pericarditis syndrome. Am J Med Genet. (2000) 91:233–6. doi: 10.1002/1096-8628(20001127)95:3<233::aid-ajmg9>3.0.co;2-3.
17. Furness L, Riley P, Wright N, Windschall D, Malattia C, Ravelli A, et al. Monogenic disorders as mimics of juvenile idiopathic arthritis. Pediatr Rheumatol Online J. (2022) 20:1. doi: 10.1186/s12969-022-00700-y
18. Hugosson C, Bahabri S, McDonald P, al-Dalaan A, al-Mazyed A. Radiological features in

congenital camptodactyly, familial arthropathy and coxa vara syndrome. Pediatr Radiol. (1994) 24:523–6. doi: 10.1007/BF02015019

19. Johnson N, Chaudhary H, Kumrah R, Rawat A, Singh S, Sharma A, et al. Syndrome of progressive deforming non-inflammatory arthritis of childhood: two patients with camptodactyly-arthropathy-coxa vara-pericarditis syndrome. Rheumatol Int. (2021) 41:1875–82. doi: 10.1007/s00296-020-04688-0
20. Kakkar RM, Soneji S, Badhe RR, Desai SB. Camptodactyly-arthropathy-coxa vara-pericarditis syndrome: important differential for juvenile idiopathic arthritis. J Clin Imaging Sci. (2013) 3:24. doi: 10.4103/2156-7514.114211
21. Ekinci RMK, Balci S, Dogan H, Yilmaz S, Aydin F, Gok F, et al. Camptodactyly-arthropathy-coxa vara-pericarditis syndrome resembling juvenile idiopathic arthritis. Mol Syndromol. (2021) 12:112–7. doi: 10.1159/000513111
22. Laxer RM, Cameron BJ, Chaisson D, Petty RE, Silverman ED, Malleson PN, et al. The camptodactyly-arthropathy-pericarditis syndrome: case report and literature review. Arthritis Rheum. (1986) 29:439–44. doi: 10.1002/art.1780290322
23. Madhusudan S, Gupta A, Prakash M, Kumar S, Agarwal V, Misra R, et al. Camptodactyly-arthropathy-coxa vara-pericarditis syndrome: a mimicker of juvenile idiopathic arthritis. Scand J Rheumatol. (2016) 45:77–8. doi: 10.3109/03009742.2015.1085085
24. Maniscalco V, Pizzetti C, Marrani E, Simonini G, Magni-Manzoni S, Ravelli A, et al. Camptodactyly–arthropathy–coxa vara–pericarditis syndrome: single case report and review of the literature. Front Pediatr. (2022) 10:981938. doi: 10.3389/fped.2022.981938
25. Marcelino J, Carpten JD, Suwairi WM, Gutierrez OM, Schwartz S, Robbins CM, et al. CACP, encoding a secreted proteoglycan, is mutated in camptodactyly-arthropathy-coxa vara-pericarditis syndrome. Nat Genet. (1999) 23:319–22. doi: 10.1038/15496
26. Murphy JM, Vanderhave KL, Urquhart AG. Total hip arthroplasty in adolescents with severe hip arthropathy associated with camptodactyly-arthropathy-coxa vara-pericarditis syndrome. J Arthroplasty. (2012) 27:1581.e5–8. doi: 10.1016/j.arth.2012.01.007
27. Nandagopalan RS, Phadke SR, Dalal AB, Ranganath P. Novel mutations in PRG4 gene in two Indian families with camptodactyly-arthropathy-coxa vara-pericarditis syndrome. Indian J Med Res. (2014) 140:221–6
28. Offiah AC, Woo P, Prieur AM, Southwood TR, Hall-Craggs MA, Manson D, et al. Camptodactyly-arthropathy-coxa vara-pericarditis syndrome versus juvenile idiopathic arthropathy. AJR Am J Roentgenol. (2005) 185:522–9. doi: 10.2214/ajr.185.2.01850522
29. Patil DV, Phadke MS, Pahwa JS, Dalal AB. Brothers with constrictive pericarditis: a novel mutation in a rare disease. Indian Heart J. (2016) 68:S284–7. doi: 10.1016/j.ihj.2016.03.020
30. Peters B, Schuurs-Hoeijmakers JHM, Fuijkschot J, Reimer A, van der Flier M, Lugtenberg D, et al. Protein-losing enteropathy in camptodactyly-arthropathy-coxa vara-pericarditis syndrome. Pediatr Rheumatol Online J. (2016) 14:1–6. doi: 10.1186/s12969-016-0093-5
31. Shashaani N, Javadi V, Rahmani K, Shiari R. Juvenile idiopathic arthritis or skeletal dysplasia: first case report of camptodactyly-arthropathy-coxa vara-pericarditis from Iran. BMC Musculoskelet Disord. 2025;26(1):802. doi:10.1186/s12891-025-09069-x
32. Shayan K, Ho M, Edwards V, Laxer R, Thorner PS. Synovial pathology in camptodactyly-

arthropathy-coxa vara-pericarditis syndrome. Pediatr Dev Pathol. (2005) 8:26–33. doi: 10.1007/s10024-004-3035-z
33. Singh S, Badiger VA, Balan S, et al. Thirteen Indians with camptodactyly-arthropathy-coxa vara-pericarditis syndrome. Clin Dysmorphol. 2024;33(4):152-159. doi:10.1097/MCD.0000000000000500
34. Sparchez M, Fodor D. Ultrasound's role in differentiating camptodactyly-arthropathy-coxa vara-pericarditis (CACP) syndrome from inflammatory arthritis in children. A narrative review. Med Ultrason. 2025;27(4):459-465. doi:10.11152/mu-4692
35. Taşar M, Eyileten Z, Kasımzade F, Uçar T, Kendirli T, Uysalel A. Camptodactyly-arthropathy-coxa vara-pericarditis syndrome. Turk J Pediatr. (2014) 56:684–6
36. Vutukuru R, Reddy KKM. Pathognomonic acetabular cysts in camptodactyly-arthropathy-coxa vara-pericarditis syndrome. Indian J Med Res. (2016) 143:834–5. doi: 10.4103/0971-5916.192082
37. Yilmaz S, Uludağ Alkaya D, Kasapçopur Ö, Barut K, Akdemir ES, Celen C, et al. Genotype–phenotype investigation of 35 patients from 11 unrelated families with camptodactyly-arthropathy-coxa vara-pericarditis syndrome. Mol Genet Genomic Med. (2018) 6:230–48. doi: 10.1002/mgg3.364
38. Zhang JM, Gao FQ, Li CF, Wang ZY, Liu YQ, Zhang YH, et al. Camptodactyly-arthropathy-coxa vara-pericarditis syndrome: the first familial case in China and novel mutations of the proteoglycan 4 gene. J Rheumatol. (2022) 49:1400–2. doi: 10.3899/jrheum.220010
